# The Diseases of Children, Their Symptoms and Treatment. A Treatise Intended for the Use of the Student and Junior Practitioner

**Published:** 1844-07

**Authors:** 


Art. VII.—*The Diseases of Children, their Symptoms and Treatment. A Treatise intended for the use of the Student and Junior Practitioner.* By G. A. Rees, m.b. Second Edition.—*London,* 1844. 8vo, pp. 300.

We have here another specimen of those *Pseudo-second editions*, which we have already more than once exposed, and which we are determined to put down. This is *no second edition*, but the identical volume issued in 1841, with a new title-page and some ten new pages of introduction, which introduces nothing. We are compelled to come to the conclusion, therefore, that the announcement of a *second edition*, under such circumstances, is, to say the least, most improper and unjustifiable. The purchasers of the former edition are warned against laying out money on a duplicate; and Dr. Rees is requested to consider, whether he is not bound in honour to state this fact in his advertisements.

